# Electronic data capture in resource-limited settings using the lightweight clinical data acquisition and recording system

**DOI:** 10.1038/s41598-024-69550-w

**Published:** 2024-08-17

**Authors:** Jakob Vielhauer, Ujjwal Mukund Mahajan, Kristina Adorjan, Christopher Benesch, Bettina Oehrle, Georg Beyer, Simon Sirtl, Anna-Lena Johlke, Julian Allgeier, Anna Pernpruner, Johanna Erber, Parichehr Shamsrizi, Christian Schulz, Fady Albashiti, Ludwig Christian Hinske, Julia Mayerle, Hans Christian Stubbe

**Affiliations:** 1 Department of Medicine II, Hospital of the LMU Munich, 81377 Munich, Germany; 2 German Center for Infection Research, Partner Site Munich, 81377 Munich, Germany; 3 Department of Psychiatry and Psychotherapy, Hospital of the LMU Munich, 80336 Munich, Germany; 4 Department of Internal Medicine II, Technical University of Munich, School of Medicine, University Hospital Rechts Der Isar, 81675 Munich, Germany; 5 Institute for Infection Research and Vaccine Development (IIRVD), Center for Internal Medicine, University Medical Center Hamburg-Eppendorf, 20246 Hamburg, Germany; 6 German Center for Infection Research, Partner Site Hamburg-Lübeck-Borstel-Riems, 20246 Hamburg, Germany; 7 Medical Data Integration Center (MeDIC LMU), Hospital of the LMU Munich, 82152 Munich, Germany; 8 Institute for Digital Medicine, Augsburg University Hospital, 86156 Augsburg, Germany; 9 Department of Anesthesiology, Hospital of the LMU Munich, 81377 Munich, Germany

**Keywords:** Clinical trial, Electronic data capture, Open-source, Progressive web app, Clinical data management, Data acquisition, Data integration, Data processing, Software, Outcomes research

## Abstract

Our prototype system designed for clinical data acquisition and recording of studies is a novel electronic data capture (EDC) software for simple and lightweight data capture in clinical research. Existing software tools are either costly or suffer from very limited features. To overcome these shortcomings, we designed an EDC software together with a mobile client. We aimed at making it easy to set-up, modifiable, scalable and thereby facilitating research. We wrote the software in R using a modular approach and implemented existing data standards along with a meta data driven interface and database structure. The prototype is an adaptable open-source software, which can be installed locally or in the cloud without advanced IT-knowledge. A mobile web interface and progressive web app for mobile use and desktop computers is added. We show the software’s capability, by demonstrating four clinical studies with over 1600 participants and 679 variables per participant. We delineate a simple deployment approach for a server-installation and indicate further use-cases. The software is available under the MIT open-source license. Conclusively the software is versatile, easily deployable, highly modifiable, and extremely scalable for clinical studies. As an open-source R-software it is accessible, open to community-driven development and improvement in the future.

## Introduction

Biomedical studies rely on methodical data acquisition and processing. EDC software is essential to biomedical research since it greatly facilitates systematic data acquisition and processing enabling meaningful analysis and relevant insights^1^. Furthermore, high-quality data acquisition and handling is the key to reliable research results. Modern EDC software must serve vastly diverse needs of biomedical studies while protecting data integrity and promoting transparent, reproducible, and reliable research. The process of data recording, using the electronic Case Report Form (eCRF) integrated into the EDC software needs to be as easy as possible, facilitating data acquisition both by study personal and by patients when filling out patient related outcome forms. In general, use of an EDC software in contrast to spread sheet solutions improves data quality whilst reducing time consumption^2,3^. The quality and comprehensiveness of data is ensured by detection of missing fields or inability to complete the submission form, a central aspect of the eCRF. An important feature of EDC software, especially with interventional studies, is the possibility of real data analysis and review, whilst monitoring the study outcome thus being able to interfere whenever a group of patients might be harmed by the intervention studied^4,5^.

Most current EDC programs are published under proprietary licenses. These include some of the most widely used EDC software in academic research, such as REDCap^6,7^. Their usage and distribution are subject to fees or specific conditions and their source code is not publicly available. Customizations and deployment of proprietary software is costly and/or bound to strict limitations. When customizations are not easily available to third parties, scientific reproducibility is impaired. Especially, data exchange and compatibility are of major importance in medical research but are often impaired by vendor-specific data architecture.

Many research groups have published innumerous studies using the REDCap software, but a head-to-head analysis against other proprietary software is not available. The most frequent burdens in the application especially involve the setup of the system since advanced computing skills are needed to run in on the local computer which often includes extra local setup fees^8–10^. Yet another popular eCRF software published under a proprietary license is soscisurvey^11^, which is mostly used for simple online surveys. It is difficult to use when more than one time point is assessed.

Important and frequent limitations of many available EDC systems are restrictions in creating versions of the database in an active study. Modifying the meta data (e.g. adding variables or visits) to a study protocol after an amendment requires archiving the existing database and a complete reset of the database.

Other commonly issues include the lack of auditability and security-concerns of the EDC software owing to many solutions being programmed rather long ago with constantly changing standards.

An important alternative to proprietary programs is open-source software. Its source code is available to the public domain under a variety of open-source licenses. Generally, open-source licenses allow the use, study, adaptation, and distribution of the source code and the software itself to everybody and for any use^12^. Some of the most widely used programming languages, software packages and operating systems such as C#, R, Ubuntu or Android are published under open-source licenses^13–17^. In the scientific context, open source-software has important advantages: publicly available source code makes open-source software transparent. Adaption and scientific analyses of open-source software are viable and not limited to license constraints. Publicly funded software development stays available to the public. Developer communities can maintain and improve the software as needed^18,19^. Unfortunately, open-source EDC programs like OpenClinica often suffer from limited features in the open-source branch^20^. Customizations and deployment of these programs require advanced software engineering skills, due to advanced programming languages and complex software architectures. Furthermore, more recent advances such as federated learning or artificial intelligence can’t be deployed.

Getting started with a clinical study might require substantial resources and time for customizing and setting up current proprietary and open-source EDC systems. For many small research projects, these resources are not affordable. In consequence, such projects do not use EDC software but rather rely on spreadsheet-programs and paper-based forms for patient questionnaires. This jeopardizes data quality and impairs collaborative multicenter research for small projects or in low-budget settings^1,21^.

To address these problems, we designed a new prototype for an open-source EDC software and an accompanying mobile client.

## Results

We created a metadata driven EDC software for clinical studies. We developed the software with the goal of creating a lightweight and scalable software, which can capture data from mobile devices and is easy to set up, manage and maintain without profound knowledge in software engineering or other significant resources. The complete source code is written in R. It is available on GitHub (https://github.com/hcstubbe/lcarsc).

### Deployment

The software can be installed as R package from CRAN or directly from GitHub using the R software package devtools (see supplementary material for detailed instructions)^22^. These methods are sufficient for installing and running the software on a local machine (e.g. a laptop or desktop computer) within a few minutes and do not require advanced IT knowledge. From here, the software can be used for a specific study on the local machine. We used this deployment strategy for the retrospective YEARS study, where we recorded the clinical data set from patient records via a single desktop computer. Alternatively, the software can be obtained as Docker image from our Docker repository and launched in a Docker container. Similarly, a local deployment on several independent machines can be created using identical configuration templates. Such deployment strategy would enable asynchronous offline data acquisition on several devices without relying on the internet or any network at all (see supplementary materiel, Fig. S1). After completion of data acquisition, the datasets of each machine are merged.

**Figure 1:**
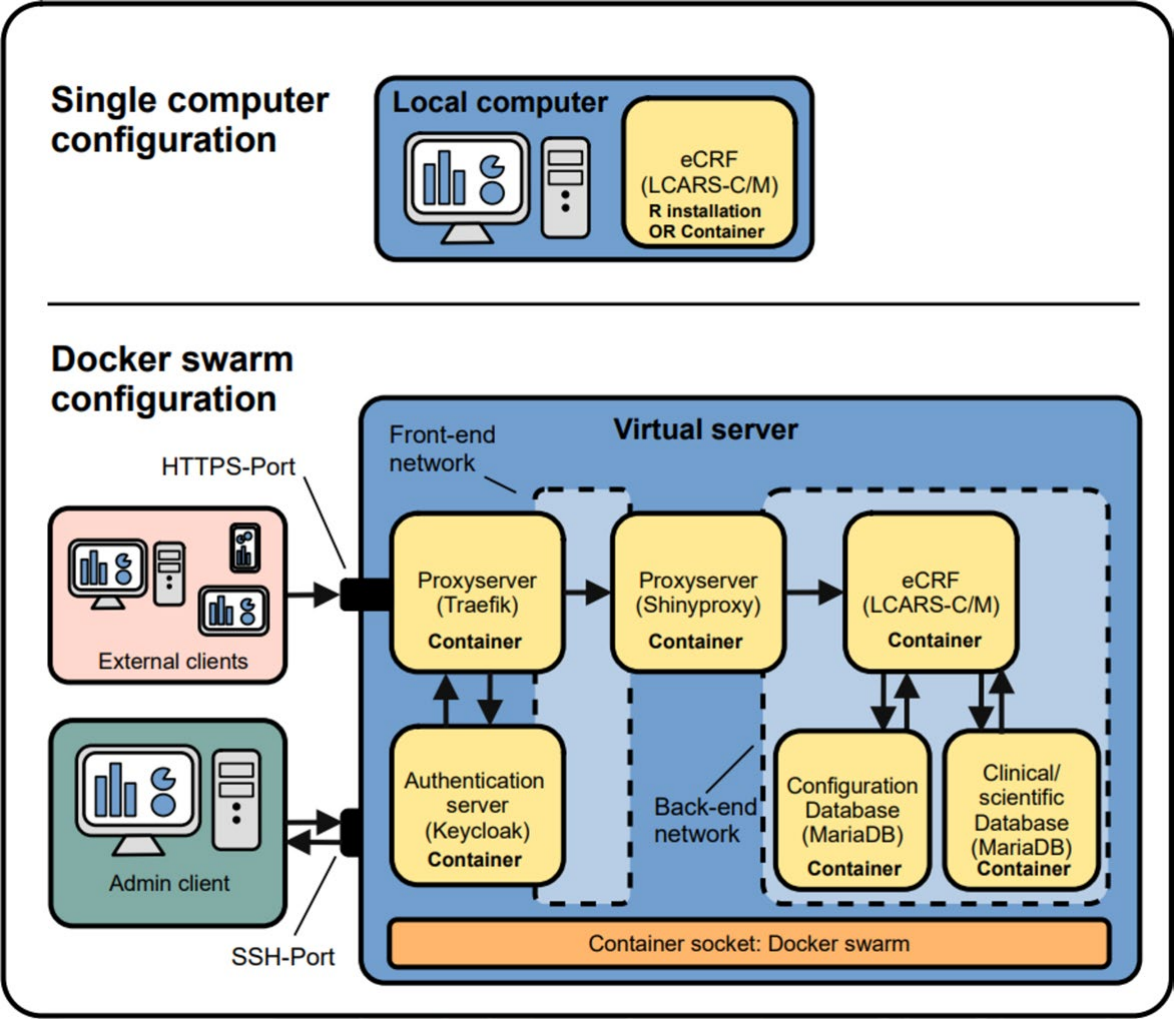
Examples for the deployment of LCARS-C and LCARS-M. The upper panel depicts a simple local deployment. The lower panel depicts a cloud-deployment using Docker swarm serving a multicenter, multiuser setting.

To deploy the software on a server, additional steps are necessary depending on the study requirements. For worldwide and Transport Layer Security (TLS) encrypted access with multiple users and secure user authentication, we use ShinyProxy: ShinyProxy is an open-source Spring boot-based web application, which deploys R/Shiny applications in docker containers^23^. This approach isolates each Shiny application in a user-specific docker container and creates an additional layer of security by separating the application management by ShinyProxy from the R/Shiny app in each container. For user authentication, we use Keycloak, which is an open-source software for identity and access management^24^. For managing web traffic and TSL certificates, we use traefik, which is a HTTP reverse proxy^25^. This setup requires a Linux (e.g. Ubuntu Server 22.04 LTS) server with at least 4 GB ram, 4 CPU cores and 50 GB disk space (preferably SSD). In addition, a DNS domain, a sub-domain for Keycloak and an e-mail address are necessary, all of which can easily be obtained from a research institution and/or a DNS domain- and e-mail-provider at no or very low cost. About one hour is needed to set up the system. This approach is summarized in Fig. 1. A detailed step-by-step description of this setup is given in the supplementary materials.

With the above deployment strategy, we are hosting a server running several studies in parallel: currently, the Post-COVID-Care Study and the URGENT-GI-Database are hosted on this server. Recently, we completed the PREDICT-COVID Study, which was hosted in parallel. Since 12/2020 until today, this server deployment was stable and we did not observe any downtime, errors, or other software-related problems.

### Designing a study

Once the study protocol of a new clinical trial is finalized and approved by all required instances, the software can be configured according to the study requirements.

**Figure 2:**
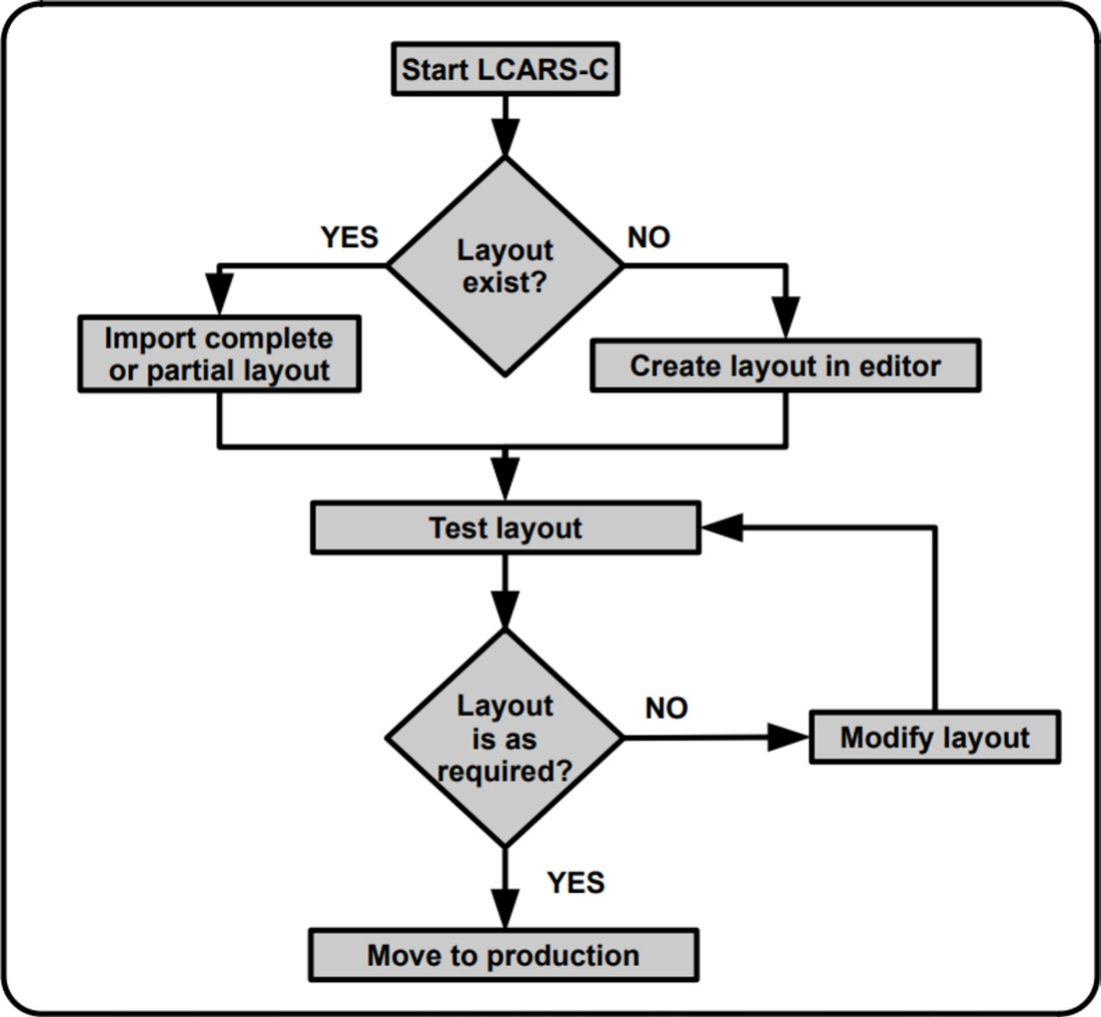
Workflow. The diagram shows the workflow for creating a new electronic case report form.

At first start, the editor mode is launched. Here, the meta data defining visits and input variables are created. After completing the process of developing and testing the meta data, is moved into deployment mode. Only in the deployment mode, clinical data is recorded permanently (Fig. 2).

**Figure 3:**
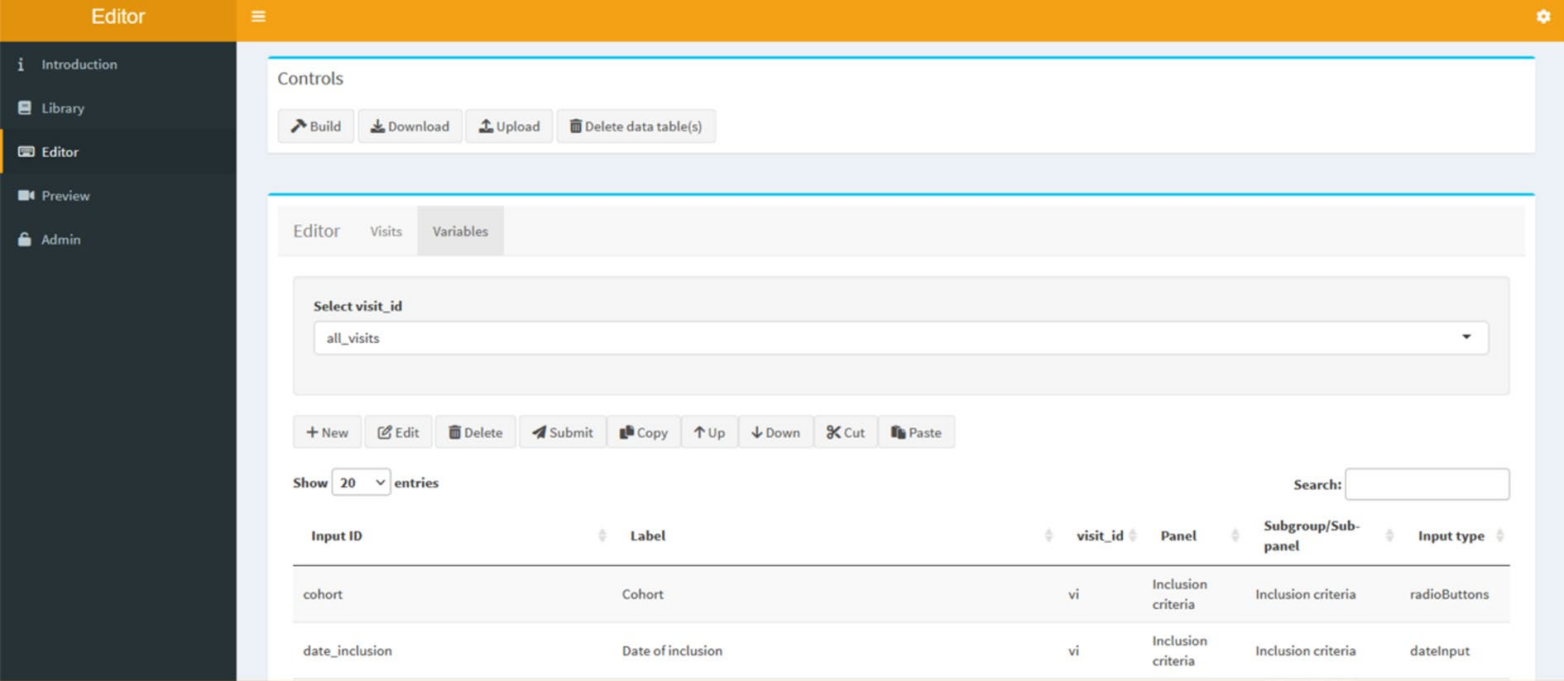
Editor user interface. interface of the widget editor. Here, widgets and visits are defined.

When first starting the meta data development, the user must define which study visits need to be recorded. For instance, the URGENT-GI-Database records a baseline visit (i.e. the hospitalization) and follow-up visits (i.e. each treatment-intervention for gastrointestinal bleeding during the hospital stay). The visits and variables are defined in the editor tab (Fig. 3). Each new visit and variable are added and edited through an input form. This form guides the process of creating new variables by allowing only correct user input and by supplying information regarding the respective input fields. If previous studies published their variable sets, these sets can be uploaded into the library. From here, required variables can be added to the respective visits. Alternatively, the complete meta data of a previous study (i.e. visit and variable definitions) can be uploaded directly into the editor creating an exact copy of the previous study or uploading previously developed definitions.

Once the required visits and variables are defined, the interface can be built in the editor tab and thereafter tested in the preview tab. If the user chooses to add a mobile visit, the mobile preview tab will show the mobile interface. If the testing meets the desired results, the application is moved into the deployment mode. Activating the deployment mode should be done with great care. In the current version, a reversal into the editor mode can only be done by the administrator. Existing visits cannot be changed anymore to protect the database integrity.

### Collecting clinical data

**Figure 4:**
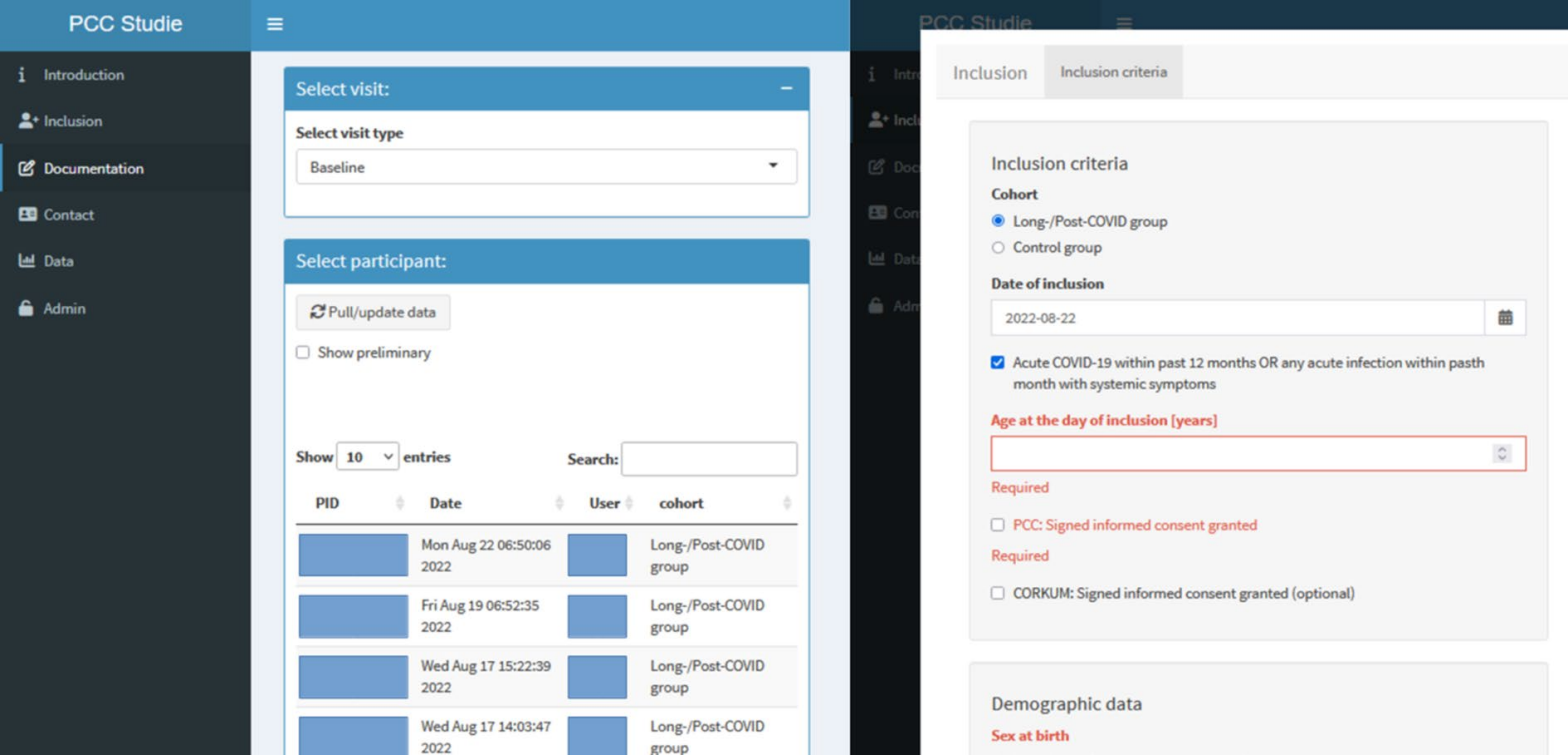
Production user interface. (Left screenshot) Interface of the eCRF in production mode. Note that the bar on top is now showing the study’s name and is colored in blue. Patient IDs and usernames are hidden; (right screenshot) an entry form showing different types of data input, such as time, text or radio buttons.

Only after activating the deployment mode, clinical data can be stored permanently. Study participants are included in the database (i.e. pseudonymized) using the inclusion tab. After inclusion, clinical data can be recorded for each participant using the documentation tab. The documentation tab provides an overview of the status of documentation for each participant (Fig. 4). The clinical data is entered and edited through an input form, which renders the required input fields for each study visit based on the respective metadata. The system supports all common data inputs: (text, integers, floats, checkboxes, time, date, radio buttons, drop-down choice menus and drop-down choice menus with search function for larger lists or vocabularies such as ICD-10 or ICHI). The input types can be extended easily if needed.

### Mobile data capture

If mobile data capture is included into the study protocol (e.g. for recording patient reported outcomes), the LCARS-M app must be deployed together with LCARS-C. LCARS-M is provided as a separate R package, to allow separate development cycles. LCARS-M must access the same databank as LCARS-C. It pulls the meta-data from the databank, which was defined by LCARS-C, to render its input interface. If LCARS-M is used by the study center to collect patient information using a mobile device (e.g. a tablet), the study personnel enters the participants pseudonymized/anonymized ID (PID) into the tablet. LCARS-M then checks, if the respective PID exists and opens the input form. If LCARS-M is configured to collect data from the same participant (e.g. from the participant’s smartphone), the participant has to login to LCARS-M using his smart phone, tablet or computer with login data provided by the study site.

### Exporting data

Once a study is completed, the complete study dataset can be downloaded as a zip file. This file contains the clinical dataset, as well as all metadata and an automatically generated codebook. In addition, the administrator can download an aggregated dataset from all visits as comma separated values (CSV) file, excel (XLSX) file, or as R Data Format (RDS) file.

### Use in clinical studies

As of today, we completed four clinical studies using the software: the prospective multicenter PREDICT-COVID-Study investigated the predictive power of the artificial intelligence (AI)-based SACOV-19 predictor and score. The retrospective YEARS-Study investigated the risk of pulmonary embolisms in patients hospitalized with acute COVID-19. The prospective Post-COVID-Care (PCC) Study investigated long-lasting signs and symptoms of COVID-19. The retrospective URGENT-GI-Database examined a large cohort of patients with gastrointestinal bleeding. For PREDICT-COVID and the YEARS-Study, two visits were recorded: one baseline and one follow-up visit. We included 124 patients in the PREDICT-COVID-Study. In total, 64 variables were collected for each study participant. In the YEARS-Study, we included 413 participants. Here, we collected a total of 101 variables. During the data capturing, we did not encounter any technical problems. Missing values in both studies were due to a lack of information in the clinical records or implausible clinical records, but never because of technical problems. Their results were published recently^26,27^. In the PCC Study, we collected up to 679 variables per participant in baseline visits and several follow-up visits encompassing medical history, current signs and symptoms, laboratory data, several questionnaires, diagnostic procedures, imaging results, specialist consultations, clinical management decisions and smart-watch data. To acquire patient reported scores and outcomes, we used the software on tablets. In total, 353 participants were included. First results of the PCC-study were published recently or are under review^28–30^. For the URGENT-GI-Study, we recorded two different study visits (one baseline and one follow-up visit). Here, we collect 173 variables per patient and included 779 participants. At the time of writing, first publications are being prepared. During all studies, no significant technical problems occurred, and no technical problems were reported by users. For all clinical studies, data was checked for consistency and completeness. Here, missing data and minor inconsistencies were due to missing or inconsistent clinical records, but never to technical issues of the software.

The ethics committee of the Medical Faculty of the LMU Munich reviewed and approved the Post-COVID-Care Study, the PREDICT-COVID-Study, the URGENT-GI-Study, and the YEARS-Study.

Several other studies are currently ongoing or in the planning stage.

## Discussion

We built an open-source software with the goal of providing an EDC system, that is easy to use, modifiable, scalable, able to capture data from mobile devices, and does not require advanced IT or software-engineering skills to get started. Since EDC software plays an essential role in biomedical research, our software facilitates transparent and reproducible research without requiring significant resources.

For scenarios, where a local computer with a single user-account is sufficient for data capture, no additional setup steps are necessary. If a more complex scenario needs to be covered, a server installation can be carried out following the deployment instructions detailed in the supplementary material. The setup does not require advanced IT or software engineering skills. The open-source software OpenClinica, for instance, requires complex setup-steps to get started with the simplest deployment method, whereas our software can be installed and is ready to go with only one R command for local deployments^20^. REDCap, a widely used EDC software, requires joining the REDCap consortium or interaction with the local REDCap informatics team, before gaining cost-free access to the software. For industry users and researchers unable or not willing to join the REDCap consortium, using the REDCap involves fees. Setting up REDCap is complex and advanced IT knowledge is recommended if REDCap is not yet available on the local research institution. Commercial offers of EDC software usually involve substantial cost for establishing these systems. Therefore, getting started with our prototype is straightforward and does not involve significant costs or other resources. A server setup can be achieved with minimal resource requirements.

The MIT license permits its use for any use case and allows studying, changing, distributing the software by anyone. Users are invited to contribute their innovations to our GitHub repository, where code changes will be reviewed and implemented based on their quality and utility. The underlying R/Shiny framework with R being an accessible and broadly used programming language among biomedical scientists, together with the modular design, set a low barrier for user contributions and code-review. Given the powerful capabilities of R in data handling, analysis, and visualization, implementing additional powerful data management and analysis pipelines seamlessly into the software can be achieved by simply adding new R/Shiny modules. In contrast, proprietary software usually does not publish or permit distributing, studying, or changing its source code. For instance, the REDCap source code is not available to the public. Modifications of REDCap, which are inspired and tested by REDCap consortium members, become property of the Vanderbilt University^6,7^. While the REDCap source code is available to consortium members, users of other proprietary software usually do not have any access to the respective source code. Compared to other EDC software, our prototype is accessible and friendly to changes by software users and other stakeholders.

Security is often brought forward to support the usage of proprietary over open-source software^7,31^. Community development and availability of source-code render open-source software more vulnerable to security issues. On the other hand, the availability of source code allows for code reviews, supporting the identification of security hazards. In addition, many open-source projects are supported by software companies. These companies implement open-source software into their own products or sell services around open-source software. Many of these products are widely and commercially used. Examples are the Linux operating system Ubuntu, which is maintained by Canonical and an open-source community and is considered extremely secure or the Docker framework, which is virtually ubiquitously used in modern web deployment. In our server deployment approach, we use enterprise-grade open-source software and a highly compartmentalized deployment strategy, isolating each user session in a single container.

The metadata driven interface and database structure along with the editor and library modules enable an uncomplicated setup for new studies. Recycling variable sets from previously published studies and importing published FHIR-conform variable sets such as the German Corona Consensus Dataset (GECCO) can greatly accelerate setting up a new clinical study reducing the required resources^32^. In addition, clinical data can be imported from FHIR-compatible servers. The usage of data standards is pivotal to guarantee data exchange and interoperability of software systems. Implementing FHIR-compatibility is a first step towards widely accepted data standards. To increase accessibility for external developers and improve integration into third-party systems, we aim to incorporate an API documentation such as the Open API standard in future releases.

In the future we aim at marking the software available for a wider use and easier deployment, where after the first setup-steps which are guided by an administrator, the conduction of the whole data input, analysis, and provision of the first figures is autonomically conducted by the software. Currently, the eCRF can be setup by the user step-by-step. We aim at implementing an automated system for data import and the import of already established questionaries. For future development we plan to implement the JSON data standard^32^ for scientific data and metadata export and import. This allows for straightforward exchange and validation of the data across systems. Furthermore, the implementation of more advanced applications, such as federated learning will be implemented.

We work to establish a community of users and contributors, allowing for long-term development and support of this open-source project. Currently, the project is being used in multiple research projects of the LMU university hospital with a growing community of users nationally and internationally.

In conclusion, we designed a new open-source EDC software, which is a versatile, easily deployable, highly modifiable, and extremely scalable EDC solution for clinical studies. We described four use cases in retrospective and prospective observational clinical studies. In these studies, we successfully tested and used the software. Several additional studies using our software are ongoing or being planned. User feedback was very positive, and no significant technical problems occurred. For the use in interventional trials, further development and certification will be required to meet FDA and EMA regulations (e.g. HIPAA or GDPR), as well as established security standards and data practices (e.g. ISO and SOC)^33^. As an open-source software and with R at its heart, LCARS-C/M is accessible and open to community-driven development, adaption, and improvement in the future.

The software is freely available under the permissive MIT open-source license.

## Methods

We aimed at making the software easy to set-up, modifiable, scalable, able to capture data from mobile devices through a mobile client and facilitate transparent, reproducible research without requiring significant resources.

### Software design

We chose the R programming language for writing the software. R is an accessible, data and statistics focused high-level programming language^17^. Unlike other popular programming languages like C++, PHP, or Java/JavaScript, it is widely used by statisticians, data scientists, in biomedical sciences and among a growing number of physician-scientist. Its capabilities in data management and analysis, as well as its wide usage among clinical scientist make it an ideal choice for LCARS-C/M: users from clinical sciences will be able to get involved in investigation, customization, and development of this software. R’s powerful data management tools provide a robust and time-tested framework to handle clinical study data.

As backbone for developing we used Shiny, an R software package for building web-based R apps^34^. It is well suited for building highly interactive web interfaces that focus on data. Developing Shiny apps is easy to learn and relies on R code not requiring classical web development languages and tools such as HTML, PHP, CSS, or JavaScript. Simple Shiny apps and modules (i.e. the building blocks of more complex shiny apps) can be coded within minutes and hours.

To enforce best practice software-design and to facilitate customization, we used the R software package golem. Golem provides a framework for building robust, production-ready Shiny apps^35^. It promotes a modular software design: specific software-tasks, such as manipulating the database or summarizing datasets are broken down into modules. Modules can be added changed, removed, and tested individually, without breaking the entire system.

Each module file entails a logic for the frontend, where the HTML output including corresponding JavaScript is defined using R code. This inhibits individual and specific frontend designs, but greatly accelerates the generation of the frontend HTML and JavaScript code. The backend logic is defined in a separate section within the module file. It contains all logic which are run on the server, such as database transactions or handling server requests and responses.

**Figure 5:**
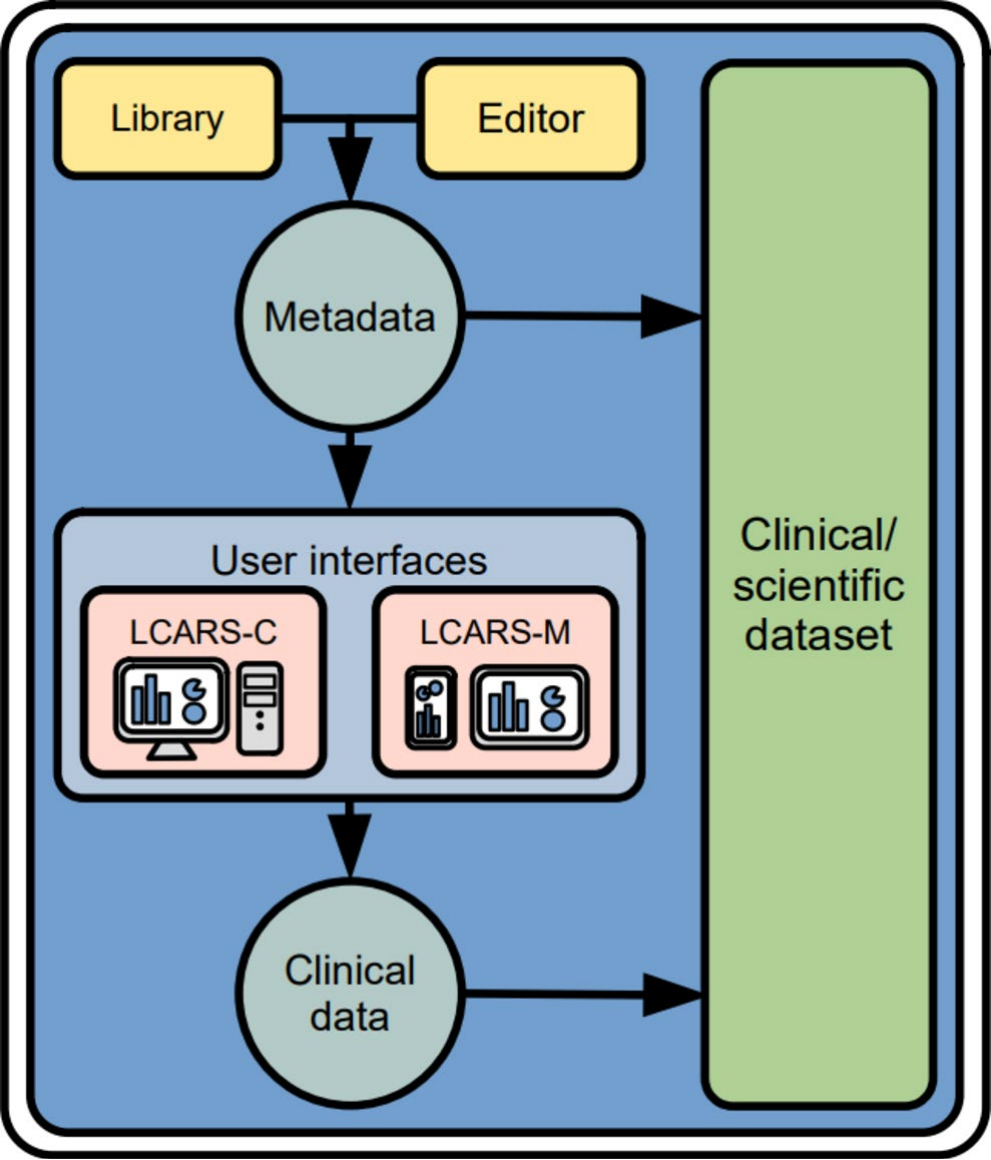
Overview. At the core of LCARS-C/M, widgets (e.g. text fields, date picker, checkboxes, etc.) are used to collect clinical data at the user interface. LCARS-C and -M generate the widgets for the user interface (both web- and PWA-interfaces) based on the widget definitions. These definitions are stored in the metadata. The metadata is created and modified by the user through the LCARS-C editor or imported from existing definitions using the LCARS-C widget library. The metadata and the clinical data collected through the user interface is stored in the clinical dataset and can be exported into different file formats.

The central functionality of the software is to capture data through a web/PWA interface by study personnel and/or study participants. We made the interface metadata driven to enable adaptions to different studies as well as systematic recording and sharing of the metadata. Metadata defines for each variable, which input widgets are displayed, how they are labeled, when and under which conditions they are shown, and what data types can be entered through them. The input forms can be organized visit-centered or participant-centered: participant information can be captured as assessed on a specific visit (e.g. a baseline visit). Alternatively, information that arises independent of study visits can be entered participant-centered (e.g. medications or diagnoses, which might change independently of programmed study visits and therefore should be independently). Metadata are managed, imported, and exported using the editor and library modules (Fig. 5), which facilitates the re-use in further project.

### Mobile data capture

To enable data capture on mobile devices, we used the R software package ShinyMobile. ShinyMobile creates a mobile focused web interface with PWA-capabilities^36^. Choosing a PWA for data input had several advantages over conventional Android iOS apps: PWAs are installable on Android devices, iOS devices and desktop computers. Installing a PWA does not require access to an app store, but simply the URL of the server hosting the PWA. We separated the mobile web interface from the core software package to allow separate development and deployment of the two components.

### Database

SQL or MySQL databases are used for storage of metadata and clinical/scientific datasets. The databases are auditable since rows are not deleted, replaced, or changed by the user. Old rows are simply marked as deleted but stay in the database. In consequence, the state of the database can be examined for any given time point. An additional module allows importing of clinical data from Fast Healthcare Interoperability Resources (FHIR) servers (e.g. clinical databases). Similarly, metadata can be imported using FIHR data formats. To allow for exchange of data with FHIR resources, we implemented a database connector for FHIR-based servers. This allows for a streamlined integration of our software alongside FHIR-based resources. We designed the database and software, so that ontologies such as ICD-10 (International Statistical Classification of Diseases and Related Health Problems, Version 10^37^), ICHI (International Classification of Health Interventions^38^) or SNOMED (widely used systematically organized computer-processable collection of medical terms^39^) can be implemented: the system supports look-up tables, where the respective dictionaries can be uploaded. Each system can then be added to the eCRF using drop-down menus with search functionality.

### Deployment strategies

To make the software scalable, we designed the software as R packages. They can be installed on a local computer running on Linux (e.g. Debian, or Ubuntu), Windows or MacOS with an installation of R. For a deployment on a server serving multiple users and locations, we tested a deployment approach with ShinyProxy, which is an open-source server software for deploying shiny apps in an enterprise grade environment^23^.

### Software verification and validation

The requirements were defined in dialogue with prospective users. The software was tested by manually examining the software’s functionality and thorough code review along with an automated unit testing strategy using the R software package testthat^40^ for automated unit testing. Frontend tests were implemented using the Selenium IDE and the Selenium WebDriver^41^.

Finally, we investigated the software’s use in four clinical studies. Data quality of these studies was assessed, and requirements of the studies were examined for their fulfillment.

### Ethical approval

All patients recruited for the studies mentioned were included in accordance with the relevant guidelines and regulations and informed consent was obtained from all subjects and/or their legal guardian(s) if necessary.

The ethics committee of the Medical Faculty of the Ludwig Maximilian University of Munich reviewed and approved the study protocols of each of the clinical studies conducted with LCARS-C.

### Role of the funding sources

The funding sources had no role in designing, data collection, analysis, interpretation, or writing.

### Supplementary Information

Supplementary Information.

## Data Availability

The source code is written in R and freely available under the MIT open-source license at GitHub https://github.com/hcstubbe/lcarsc. The datasets generated in the studies described are available from the corresponding author on reasonable request.
