# Supplementary material for: Electronic data capture in resource-limited settings using the lightweight clinical data acquisition and recording system
Source: Sci Rep. 2024 Aug 17;14:19056. doi: 10.1038/s41598-024-69550-w (PMC11330438; doi:10.1038/s41598-024-69550-w)
Supplement: Supplementary file 1 — Supplementary Information. [file 41598_2024_69550_MOESM1_ESM.pdf]

Electronic data capture in resource-limited settings using the lightweight clinical data acquisition and recording system for clinical studies – LCARS-C

Supplementary material

Table of content

Figure S1 ……………………………………………………………………………………………………….. 1

Examples of LCARS-C deployment……………………………………………………………………………… 2

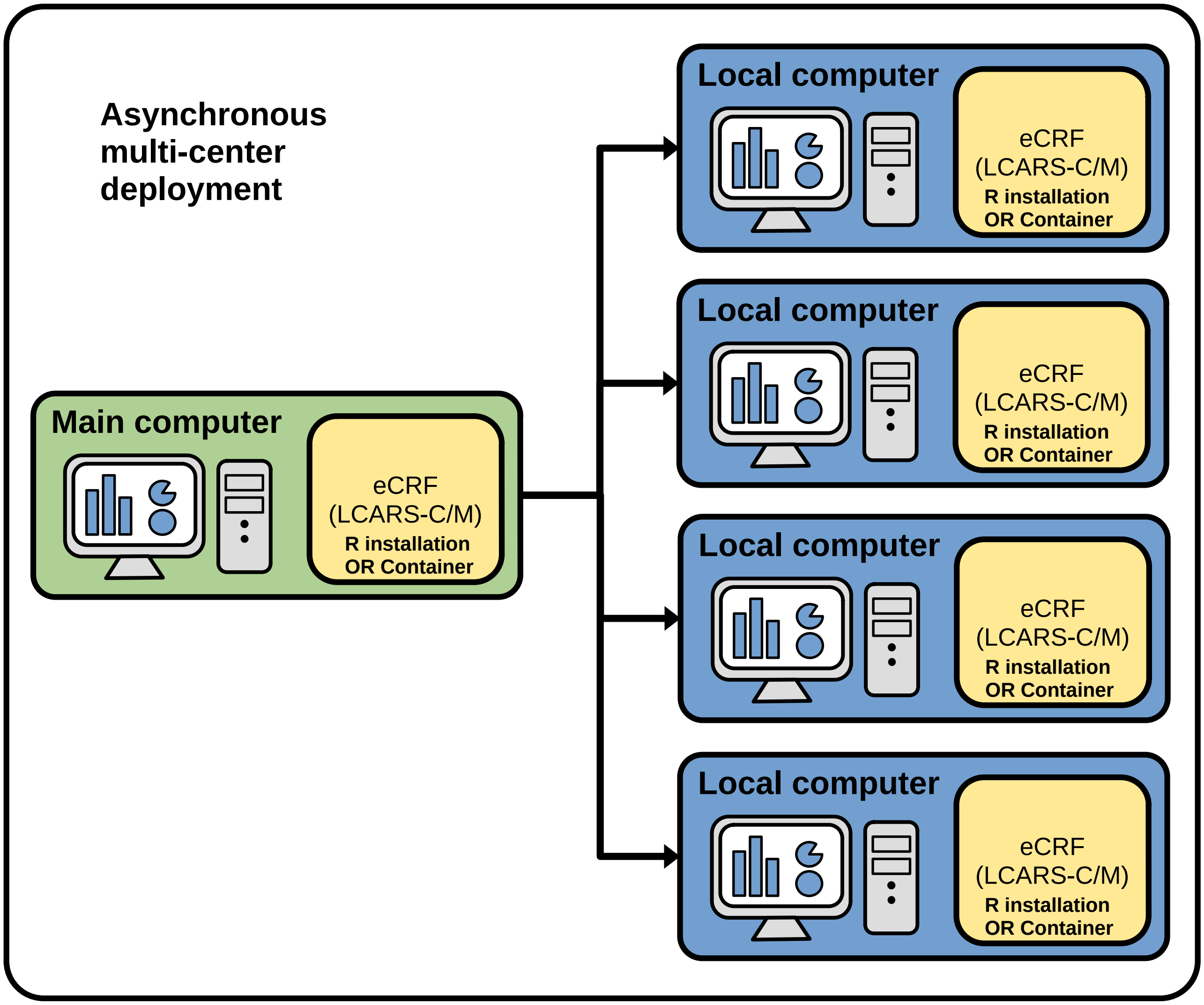

Figure S1. Asynchronous deployment. Each instance is installed locally. The metadata is developed in the main computer and shared with all local instances. After completion of data capturing, all datasets are combined. Each local instance is identified by a unique identifier which specified in the editor.

Examples of LCARS-C deployment

A1: Local deployment with R/R Studio

Installation

Install R and R Studio. Clone this repository, open R Studio, and install LCARS-C from the repository's base folder including the lcarsM dependency:

```
devtools::install_local("dependencies/lcarsM.tar.gz")
devtools::install_local()
```

Example

```
library(lcarsc)
lcarsc::run_app(ecrf_database_driver = RSQLite::SQLite(),
        ecrf_dbhost = "dbtest",
        ecrf_dbname = "db_test_data.sqlite3",
        config_database_driver = RSQLite::SQLite(),
        config_dbhost = "dbtest",
        config_dbname = "db_test_cfg.sqlite3",
        options = list(host = '0.0.0.0', port = 3838))
```

A2: Local deployment using with MariaDB and Docker

This deployment starts a single app and makes the user interface available in the local network.

Install Docker.

Run the following in this respository's base folder. The database data is saved in **./database/data**.

```
# Start
docker-compose -f ./shinyproxy/app_and_db.yml up

# Shut down
docker-compose -f ./shinyproxy/app_and_db.yml down
```

A3: Local deployment using ShinyProxy and MariaDB with Docker

This can be used as a template for local deployments.

- Install the Docker engine
- Clone this repository and set the repository's folder as working directory.
- Create folders for database permanent storage:

```
mkdir -p ./shinyproxy/mariadb/logs
mkdir -p ./shinyproxy/mariadb/data
```

- Start the shinyproxy using docker-compose (the docker images are pulled automatically):

```
export DOCKERID=$(getent group docker | cut -d: -f3)
docker-compose -f ./shinyproxy/shinyproxy_local.yml up
```

- Open link to access shiny proxy (use test_user as username and password; see application_test.yml file): http://localhost:7070/login

Backup and restore MariaDB database

- Backup:

```
docker exec CONTAINERID /usr/bin/mysqldump -u root --password=coucoutest mydbtest > backup.sql
```

- Restore:

```
cat backup.sql | docker exec -i CONTAINERID /usr/bin/mysql -u root --password=coucoutest
mydbtest
```

B: Web deployment with Docker swarm

This deployment strategy is based on this tutorial.

Please also check out this repository on GitHub for the latest deployment strategy.

As prerequisites, you need

- A server with 4 cores, 8 GB memory, and min. 50 GB SSD storage running Ubuntu 20.04 LTS or later, and administrator access to the server
- A domain (here referred to as [FQDN]) with the following sub-domains:
    - traefik.[FQDN]
    - keycloak.[FQDN]
    - study.[FQDN]

Setup server

- Install Docker and join user into Docker group

```
sudo apt install docker.io
sudo systemctl enable --now docker
sudo usermod -aG docker [USER NAME]
```

- Re-login
- Pull Docker images

```
docker pull hstubbe/lcarsc:latest
docker pull mariadb
docker pull traefik
docker pull openanalytics/shinyproxy
docker pull quay.io/keycloak/keycloak
```

- Setup Docker swarm

```
docker swarm init
```

- Get tokens

```
docker swarm join-token worker
docker swarm join-token manager
```

- Create Docker networks

```
docker network create --driver=overlay sp-net
docker network create --driver=overlay traefik-public
```

- Setup traefik

```
export NODE_ID=$(docker info -f '{{.Swarm.NodeID}}')
export EMAIL=[VALID E-MAIL]
export DOMAIN=traefik.[FQDN]
export USERNAME=admin
export HASHED_PASSWORD=$(openssl passwd -apr1)
docker node update --label-add traefik-public.traefik-public-certificates=true
$NODE_ID

curl -L dockerswarm.rocks/traefik.yml -o traefik.yml
docker stack deploy -c traefik.yml traefik
docker stack ps traefik
docker service logs traefik_traefik
```

- Get this repository

```
git clone https://github.com/hcstubbe/lcars_webhosting.git
```

- Setup database

```
sudo mkdir -p /data/study/mariadb/ecrf/data/ /data/study/mariadb/ecrf/logs/
docker stack deploy -c shinyproxy/mariadb.yml db
```

- Updated keycloak theme (if changed)

```
docker build -t keycloak:updated_theme keycloak/.
```

- Deploy Keycloak

```
export KEYCLOAK_DOMAIN=keycloak.[FQDN]
docker stack deploy -c shinyproxy/keycloak.yml keycloak

export APP_DOMAIN=study.[FQDN]
export DOCKERID=$(getent group docker | cut -d: -f3)
docker stack deploy -c shinyproxy/shinyproxy.yml shinyproxy
```

After deploying keycloak, you need to configure keycloak as follows:

- Add a new Real by clicking “Add Realm” and select a name
- Go to “Clients” on the side bar and click “create” and select a name for the client application.yml (i.e. the ShinyProxy server)
- On the main Settings page:
  - Turn “Authorization Enabled” on
  - Add https://your.domain.org/* to “Valid Redirect URIs”

- Click “Save”

- Click the “Credentials” tab in the top tab bar and copy the Secret. This secret need to be added to the ShinyProxy application.yml
    - Create users:
    - Click “Users” in the left sidebar. Create new user. Add a user name AND a “last name”. The last name can be identical with the username. The last name will be displayed in the LCARS-C/M user-interface and is required.
    - Click “Save”
    - Click the “Credentials” tab and enter a temporary password
    - Create a role for the user. The role names need to be specified in the ShinyProxy application.yml, too. The roles control, which application each user can access. The admin role should only be awarded to the system admin.

- Deploy ShinyProxy

```
export APP_DOMAIN=study.[FQDN]
export DOCKERID=$(getent group docker | cut -d: -f3)
docker stack deploy -c shinyproxy/shinyproxy.yml shinyproxy
```

Backup database

- Create backup

```
docker exec CONTAINERID /usr/bin/mysqldump -u root --password=[YOUR PASSWORD] db > backup.sql
```

- Restore

```
cat backup.sql | docker exec -i CONTAINERID /usr/bin/mysql -u root --password=[YOUR PASSWORD] db
```

- Copy backup to local machine

```
scp [user]@[IP]:~/servername/backup.sql ~/backup.sql
```

Remove stacks

If you wish to stop the server use the following:

```
docker stack remove shinyproxy
docker stack remove keycloak
docker stack remove traefik
docker stack remove mariadb
docker network prune
```
